# Supplementary material for: The causal role of circulating inflammatory markers in osteoporosis: a bidirectional Mendelian randomized study
Source: Front Immunol. 2024 Jul 18;15:1412298. doi: 10.3389/fimmu.2024.1412298 (PMC11291241; doi:10.3389/fimmu.2024.1412298)
Supplement: Supplementary file 1 [file DataSheet_1.zip › supplementary material/CIM-OSTEOPOROSIS /GCST90274833.csv_forest.pdf]

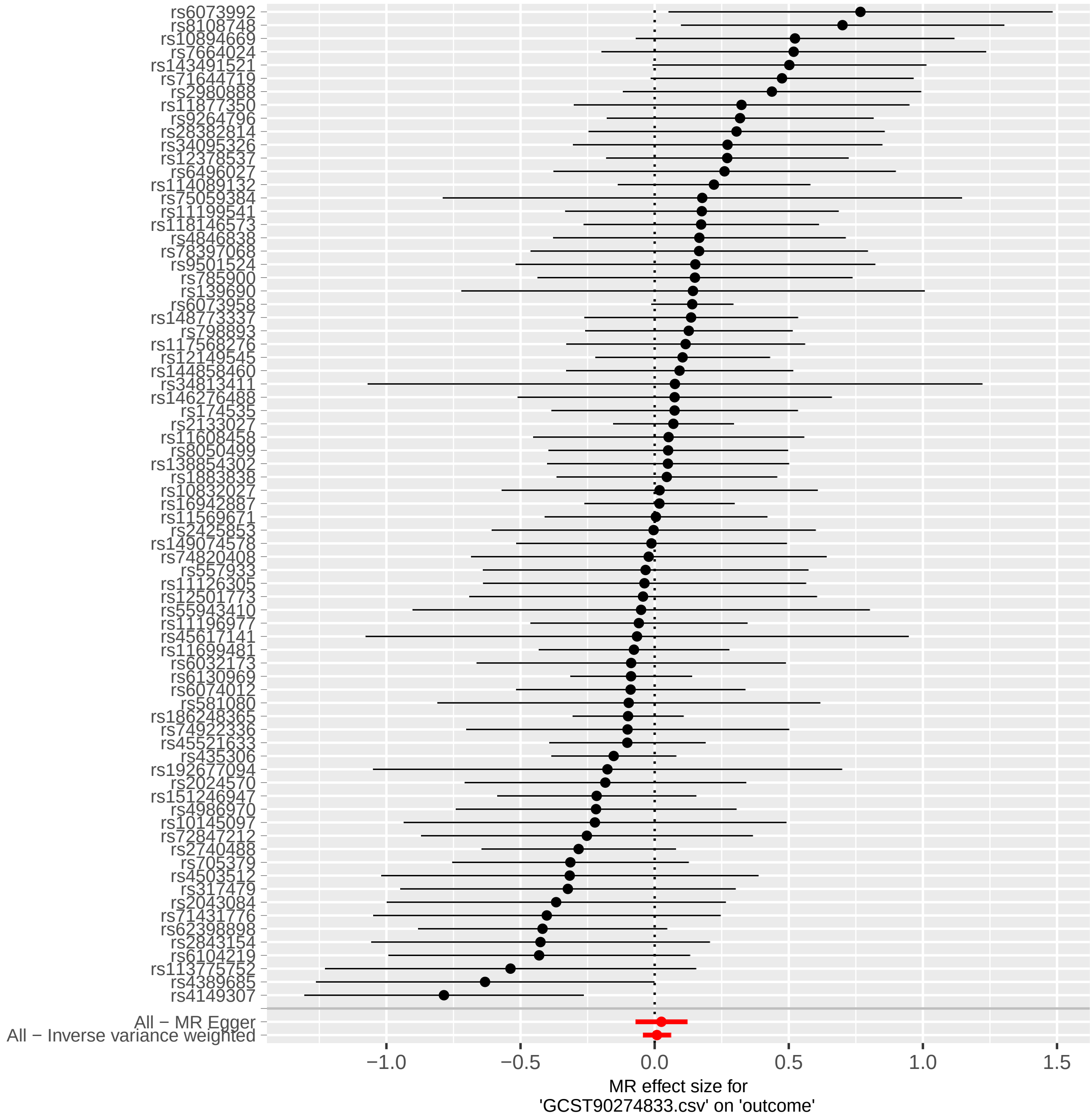
rs6073992
rs8108748
rs10894669
rs7664024
rs143491521
rs71644719
rs2980888
rs11877350
rs9264796
rs28382814
rs34095326
rs12378537
rs6496027
rs114089132
rs75059384
rs11199541
rs118146573
rs4846838
rs78397068
rs9501524
rs785900
rs139690
rs6073958
rs148773337
rs798893
rs117568276
rs12149545
rs144858460
rs34813411
rs146276488
rs174535
rs2133027
rs11608458
rs8050499
rs138854302
rs1883838
rs10832027
rs16942887
rs11569671
rs2425853
rs149074578
rs74820408
rs557933
rs11126305
rs12501773
rs55943410
rs11196977
rs45617141
rs11699481
rs6032173
rs6130969
rs6074012
rs581080
rs186248365
rs74922336
rs45521633
rs435306
rs192677094
rs2024570
rs151246947
rs4986970
rs10145097
rs72847212
rs2740488
rs705379
rs4503512
rs317479
rs2043084
rs71431776
rs62398898
rs2843154
rs6104219
rs113775752
rs4389685
rs4149307
All − MR Egger
All − Inverse variance weighted
−1.0
−0.5
0.0
0.5
1.0
1.5
MR effect size for
'GCST90274833.csv' on 'outcome'
